# Development of a Low-Power Underwater NFC-Enabled Sensor Device for Seaweed Monitoring

**DOI:** 10.3390/s21144649

**Published:** 2021-07-07

**Authors:** Caroline Peres, Masoud Emam, Hamed Jafarzadeh, Marco Belcastro, Brendan O’Flynn

**Affiliations:** Tyndall National Institute, University College Cork, T12 R5CP Cork, Ireland; masoud.emam@tyndall.ie (M.E.); hamed.jafarzadeh@tyndall.ie (H.J.); marco.belcastro@tyndall.ie (M.B.); brendan.oflynn@tyndall.ie (B.O.)

**Keywords:** aquaculture, sensor system, data logger, seaweed monitoring, inertial motion unit

## Abstract

Aquaculture farming faces challenges to increase production while maintaining welfare of livestock, efficiently use of resources, and being environmentally sustainable. To help overcome these challenges, remote and real-time monitoring of the environmental and biological conditions of the aquaculture site is highly important. Multiple remote monitoring solutions for investigating the growth of seaweed are available, but no integrated solution that monitors different biotic and abiotic factors exists. A new integrated multi-sensing system would reduce the cost and time required to deploy the system and provide useful information on the dynamic forces affecting the plants and the associated biomass of the harvest. In this work, we present the development of a novel miniature low-power NFC-enabled data acquisition system to monitor seaweed growth parameters in an aquaculture context. It logs temperature, light intensity, depth, and motion, and these data can be transmitted or downloaded to enable informed decision making for the seaweed farmers. The device is fully customisable and designed to be attached to seaweed or associated mooring lines. The developed system was characterised in laboratory settings to validate and calibrate the embedded sensors. It performs comparably to commercial environmental sensors, enabling the use of the device to be deployed in commercial and research settings.

## 1. Introduction

In aquaculture, maintaining the health and welfare of livestock, optimising resources, and sustainable growth are the current challenges in the continued efforts to balance economics and environmental sustainability in the farming practices of the future. One promising solution is to adopt the practice of integrated multi-trophic aquaculture (IMTA) [1,2]. IMTA cultivates different marine species in the same site, taking advantage of using by-products (such as waste and uneaten food) from one species as inputs (fertilizer, food, and energy) for the growth of other species. This is more sustainable than monoculture aquaculture sites, due to its lower environmental impact, product diversification, spatial optimisation, and better management practices [1,2]. However, to optimise the production and management system, and to understand how the species interact with each other and with the environment, technology to remotely monitor environmental and biological conditions is needed.

A review of optical remote monitoring techniques for kelp is described in Shroeder et al. [3] where the authors describe methods that are useful for determining biomass and temporal trends of kelp communities. These techniques involve image acquisition of an area to detect floating macroalgae, and different species have different spectral responses to image sensors due to their different morphology and colour. The authors highlight the need for ground-truthing the data and the need for image processing algorithms that can be computationally expensive. Another review of remote monitoring methods is described in Bennion, Fischer, and Brodie [4], and it includes other techniques such as monitoring via Lidar and Sonar.

The sensing techniques described in these papers are useful to monitor large areas for macro-algae distribution and to indirectly derive the biomass in the area. However, the spatial resolution of these techniques is limited, as an aquaculture site can be covered by just a few pixels in the image. Ottinger, Clauss, and Kuenzer [5] argue that very high resolution sensors are better suited for aquaculture site mapping and monitoring.

Another potential issue with these optical remote monitoring techniques is the over- or underestimation of the monitored variables. For example, Meneghesso et al. [6] identified a mismatch between the remote monitoring and deployed in situ temperature loggers. Other studies [7,8] found the same problem. This highlights the need for an in situ deployed sensor to accurately monitor variables such as temperature.

Different abiotic and biotic factors affect the aquaculture farm production. In particular, wave exposure and water motion affect the growth of seaweed, but the effect is not completely understood [9,10,11,12]. Since most studies of this effect use non-direct measurements of wave exposure, such as wave exposure index [13] derived by wind speed and incidence, they do not provide good correlation with actual wave measurements [14].

The accurate measurement of these environmental parameters is needed to design and validate ecosystem models for IMTA production, such as Fan et al. [15], due to the current challenge of IMTA in understanding how the species interact with each other and the environment. To validate these models and improve production efficiency, environmental sensors—such as temperature, light, water quality (such as the concentration of dissolved oxygen, water pH, salinity, turbidity, concentration of pollutants), nutrient (dissolved nitrogen) availability, and water motion—are needed [16,17].

Monitoring of water quality and some environmental parameters—such as temperature—in an aquaculture setting is well supplied by commercial off-the-shelf sensors. Their measurement parameters include temperature, light radiation, and water characteristics (dissolved oxygen, pH, salinity, nitrogen concentration). However, these sensors are expensive and usually not co-located with the seaweed growth site. Additionally, these sensors are usually big, require cables and a power supply, and measure only one parameter, which increases the power consumption, maintenance, and cost [18,19]. For example, Visch, Nylund, and Pavia [10] used Onset HOBO Pendant UA-002-64 loggers [20] to monitor and log temperature. However, this commercial sensor is big, only measures temperature and light, and needs to be wired which limits the deployment in areas where the accessibility is hard or there is no power.

In the case of measuring of wave exposure and water motion rates, sensors such as acoustic doppler current profilers (ADCP) or water buoys are generally used. However, these sensors are usually expensive and do not allow for multiple point deployments. Some cheaper solutions were developed by [14,19,21,22] using accelerometers, but none of them provide a complete multi-sensor integrated solution.

Judge, Choi, and Helmut [23] review the current logger technology for intertidal environments. Although not the same application, the environment is similar to the one seaweed is cultivated, and the loggers used to monitor are the same. They argue that the current loggers available are limited in: (1) construction—as the attachment requires large amounts of epoxy that interfere with temperature readings; (2) lack of real-time data due to the wireless communications constraints of underwater environments and hard to download data; (3) miniaturization. The dataloggers used in the studies compiled by Judge, Choi, and Helmut [23] are: (a) iButton devices by Maxim Integrated [24] that, although developed for harsh marine environment, require a cable to transmit data to a receptor; (b) HOBO TidbiT by Onset [25] that is small and waterproof. Both solutions, however, only log temperature.

Other sensors to monitor aquaculture species were developed using easy-to-use platforms such as Arduino [26,27]. The device developed by Beddows and Mallon [27] is able to log only temperature with an operational lifetime of up to 1 year in non-rechargeable batteries. The device is housed in a big enclosure that is able to withstand the harsh marine environment. The data collected need to be downloaded by recovering the device.

For wave measurement, Knight et al. [26] developed an IoT-enabled tide gauge with a pressure sensor and an Arduino. This sensor is designed to be placed in a fixed position and requires cables to a power source. Kennedy et al. [22] developed a miniature wave measurement device as a replacement for wave buoys. It has a 6-degrees-of-freedom (DoF) inertial measurement unit (IMU) and a Zigbee wireless connection.

All the solutions discussed above have some problems in common, with varying degrees: big size that limits the deployment method; power requirements such as cables, non-rechargeable batteries, external power supplies; data recovery, as most do not transmit data wirelessly (requiring cable loggers that store data for transmission) or limit deployment in hard-to-reach locations, as device recovery is challenging; limited to one sensor type per enclosure—as far as we know, no miniaturised multi-sensor modality solution is available for seaweed monitoring.

In this work, we present a novel miniature low-power near-field communication (NFC)-enabled data acquisition system to monitor aquaculture species. This sensor system monitors temperature, light intensity, depth, and motion, logs the data internally, and can transmit the logged data via NFC (to a smartphone, for example). It also has an internal machine-learning-enabled microcontroller, which can be used to analyse data internally.

The mechanical enclosure design allows it to be attached to IMTA species for data acquisition. The device is designed to be attachable to seaweed and kelp blades or stipes: it has a texture on the bottom side for gluing onto the blades; it also has holes for threading safety threads to secure the device to the mooring line or to tie it to the stipe. The temperature and depth sensor has a direct interface with water, which allows the sensor to accurately measure water temperature even with glue or epoxy attachments.

The sensor device can communicate with NFC-enabled readers (such as smartphones) to configure the sensors with custom sampling frequencies, communicate status, and to download data.

An IP-rated enclosure for the multi-modal sensing system was developed, and the device was characterised in lab experiments to verify if it complies with the requirements outlined above and to determine its battery lifetime with different sensor sampling conditions prior to deployment.

This paper is organised as follows: In Section 2, we present the design rationale and architecture of the device. We explain in detail the hardware, enclosure, antenna, firmware, and smartphone application developed. In Section 3, we present the methods and results for the characterisation of the sensors embedded in the device, and the power consumption measurements. In Section 4, we discuss the results and the relevancy of the sensor device developed for the aquaculture industry.

## 2. Materials and Methods

### 2.1. Hardware Architecture

In conjunction with stake holders and end users (seaweed farm owners) and knowledge from current state-of-the-art, the requirements for the device were defined as follows:

- The device must be as small as possible as to not disturb the seaweed physiology; be reusable to reduce environmental waste and pollution; waterproof and resistant to the marine environment.
- The sensors should be configurable regarding sampling frequency and other relevant parameters to allow greater customisation in the deployment. The device should be able to log temperature, depth, movement via acceleration, and light intensity on a single small package.
- The device must have a battery and wireless communication to be able to collect the logged data remotely.
- The battery should be rechargeable to reduce waste, and its lifetime should be maximised to allow longer periods of deployment without the need for farm operator interaction.

**Figure 1:**
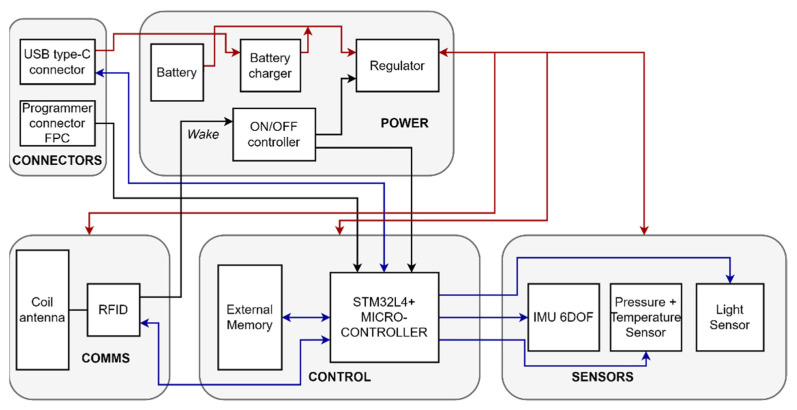
System block diagram for the device hardware.

Based on the user requirements above, the device developed is composed of various sensor modalities, a microcontroller as a supervisor for the device, an external flash memory to save collected data, and a radio-frequency identification (RFID)/NFC front-end to communicate with NFC readers while deployed. The system block diagram for the device is shown in Figure 1.

When selecting the components for the system, the main constraints were size, power consumption, and compatibility with the other components and the system main voltage value.

Due to the size constraints identified and the extended operational lifetime requirement, the battery technology chosen was lithium-ion polymer (LiPo). The additional benefit of this type of battery is the small weight and the rechargeability. The nominal voltage of a LiPo battery cell is around 3.7 V, so it can be used to power integrated circuits in the 3.0–3.3 V range. We chose 3.0 V as the main voltage of the circuit to extend the power-on time, letting the battery discharge as much as possible. The battery chosen has a management system embedded that protects the battery from over-discharge, overcharge, and short circuits. It is a 300 mAh capacity LiPo battery with dimensions of approximately 36 mm × 12 mm × 6.5 mm.

A battery charger IC was added to the design to control the charging of the battery over the USB connector. The IC used for this is has a small footprint, allows the configuration of the charging current via an external resistor connected to one of its pins, and it has a pin input for a thermistor for thermal shutdown in case of high temperature sensed.

The battery is always connected and powers the whole system via a low-dropout regulator (LDO) when in normal and sleep modes. To conserve battery energy when not in the data collection mode, the device has a shutdown mode that supplies power only to the wake-up subsystem.

An ON/OFF controller [28] was added to the design to manage the shutdown mode by turning on and off the main system LDO. The controller turns on the main system power regulator via two events: (1) a wake-up signal from the NFC application or (2) by connecting the device to a USB host.

The sensors chosen for the device were carefully selected to be as small form factor as possible, with very low power consumption and compatibility with the system voltage of 3.0 V. Another factor that went into the sensor selection is the configurability and resolution.

The movement sensor used in the system integrates a 3D accelerometer and a 3D gyroscope in a very small package with very low power consumption and low noise. The IC also has an embedded machine learning core that can be used to filter and detect features in the motion data, reducing the processing needed in the controller.

For temperature and pressure sensing, the device chosen has a very small form factor and is optimised for water depth measurements. It has a high depth resolution of 0.2 cm in water, operating pressure range of 3 MPa, and low power consumption. The pressure sensing opening is built for chemical endurance in hash liquid media (such as seawater), and it can be watertight using an O-ring. In addition to pressure sensing, it provides temperature sensing as well ranging from −20 °C to 85 °C and resolution of 0.0022 °C.

For light/lux sensing, a colour + clear light sensor with embedded IR (infrared) blocking filter was selected. A colour sensor was preferred due to the possibility of correlating the RGB channel values with the photosynthetically active radiation (PAR) sensors, in addition to the clear (white light) channel that correlates with the lux values. The sensor chosen has programmable integration time and gain, which allows it to measure values in different light intensity environments. Other key factors are the very small size and the low power consumption.

A microcontroller (MCU) was added to the design to control and manage the whole system, transfer data from sensors to external memory, and communicate with hosts. The requirements for the MCU include low power consumption, small size, high processing power, large RAM, enough peripherals for all the sensors and external circuits, and floating point. The MCU chosen was the STM32L4R5QII6 [29], due to its low power, machine learning compatibility, and high CoreMark and ULPMark scores [30,31] compared to other MCUs of same power consumption.

To make sure there is enough space to save all sensor data collected, the SPI Flash memory with the largest memory size possible was selected. For a serial SPI Flash, the largest memory capacity available in a commercial off-the-shelf IC is 1 GB. This is roughly equivalent to 2 weeks of continuous accelerometer and gyroscope data (with sampling frequency of 52 Hz).

To communicate download data collected and wake-up the system, two communication interfaces are present in the device design: (1) a wired interface using a USB type C connector and (2) a wireless communication interface using NFC/RFID at 13.56 MHz.

The USB connection can be used to communicate with the MCU, download data, configure the system, update the firmware, and wake-up the system. USB was chosen because of its ease of use and compatibility with any personal computer and laptops. The USB connector selected is waterproof with an O-ring around its external chassis, which adds another layer of protection to the device when using it in wet areas. The USB is also used to provide the power to the device, powering the main system voltage and/or charging the battery.

Water, especially seawater, is a challenging environment for wireless communication [32]. Radio-frequency (RF) communications suffer from propagation loss due to the water salinity, and this effect is directly correlated with the frequency of transmission as the higher salt concentration makes the water conductive in these high frequencies [33,34]. On the other hand, lower frequencies—which would be less affected by the attenuation—require large antennas [34]. To mitigate this problem, one can use electromagnetic fields in the near-field (the transmission principle behind some RFID tags and near-field communication (NFC) devices), also called magnetic induction (MI) [35,36]. These systems transmit using the magnetic field, which suffers less attenuation in seawater than the electric field used in other RF communications [35]. The lower frequency of these systems also lowers the attenuation factor [36].

Near-field communication (NFC) is a standard of communication on the high-frequency (HF) RFID at 13.56 MHz. In an RFID system, the transmitter (also called reader) generates an electromagnetic field from its loop antenna that induces a voltage in the receiver (or tag) antenna located inside this field. The reader-tag pair then works like two coupled inductors, and the impedances at each side are reflected back to the opposite side, which is how data are transmitted [37]. An added benefit of this system is that the induced voltage in the antenna can be used to power up the tag, effectively harvesting energy from the field.

The NFC standard is widely used today, and a great number of modern smartphones have an NFC reader/writer controller embedded to be used as a smart wallet. This can then be leveraged to be an easily accessible and operable reader for the device through the development of suitable smart phone apps. Another benefit is that the NFC reader provides most of the power used in the communication, as stated before, which greatly reduces the power consumption for this communication interface. On the other hand, the range of NFC is limited by the reader transmitted power and antenna sizes [36,38]. The NFC standard [39] limits the strength of the magnetic field created by a reader, effectively limiting the range for commercial systems to around 20 cm.

The interface for the NFC chosen was the ST25DV chip transponder [40], which on top of having an internal EEPROM, can also communicate via a mailbox system that can transmit up to 256 bytes per message on demand. This makes the communication between reader and transponder more efficient, as there is no need to read/write the internal EEPROM. The ST25DV chip is connected to the MCU via I2C serial interface, allowing it to act as a bridge between the MCU and the NFC reader.

This chip can also communicate with the NFC reader battery-less. This means that the system can be in shutdown mode and still be able to respond to commands. We use this feature to create a wake-up circuit that the NFC chip turns on when receiving a command from the reader. In this mode, the battery only powers the real-time clock in the MCU and the wake-up circuit, effectively reducing energy consumption when not in use.

**Figure 2:**
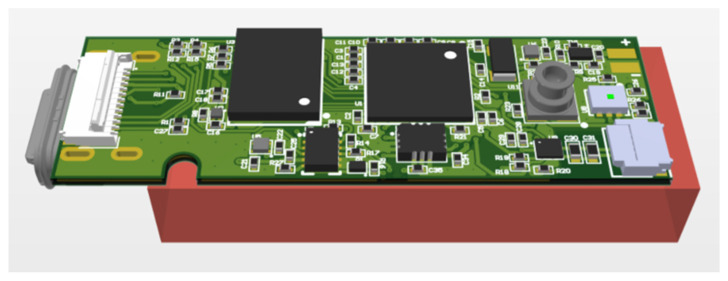
3D view of the final designed PCB. The orange block represents the battery.

The electronics are mounted on very small custom-fabricated printed circuit boards (PCB) with 4 layers and impedance matching for the USB line. Figure 2 shows the final PCB design.

### 2.2. RFID/NFC Antenna Design

**Figure 3:**
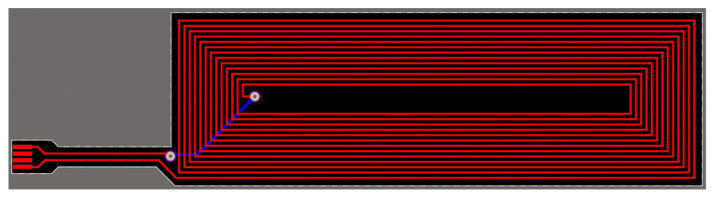
Flexible PCB track design for the antenna resonant to 13.56 MHz when combined with the ST25DV NFC chip (L = 4.88 µH).

We designed a custom antenna for this application, as no COTS antenna had the size required for the device. The antenna was designed to be a flexible printed circuit board that connects to the main PCB via a flexible printed circuit (FPC) connector. Its size (excluding the connection cable) is 40 × 13 mm and was chosen due to the overall PCB and battery size. The antenna parameters were designed using ST Antenna eDesign Suite [41] to match the expected impedance for the NFC chip used in the design (ST25DV). To design the antenna and calculate its parameters, the procedure in STMicroelectronics application note AN2866 [42] was followed. Figure 3 shows the pattern design for the rectangular antenna flexible PCB.

The internal capacitance of the NFC chip used (ST25DVxxx) for the antenna pins is 28.5 pF. To make a resonant antenna at the 13.56 MHz frequency, we can use the following relationship to determine the inductance of the antenna:

$$X=2\pi fL+\frac{1}{2\pi fC}=0$$

From the above equation, we calculate the required antenna inductance to be *L* = 4.83 µH. Using the ST Antenna eDesign Suite, the parameters for conductor width and spacing were calculated for the antenna size, but due to the small size, a compromise was needed on the inductance value. The real inductance calculated was *L* = 4.88 µH. To solve the discrepancy in inductance value and possible fabrication deviations, the main PCB was designed with space to solder a capacitor in parallel with the antenna connector to complete the resonant circuit.

**Figure 4:**
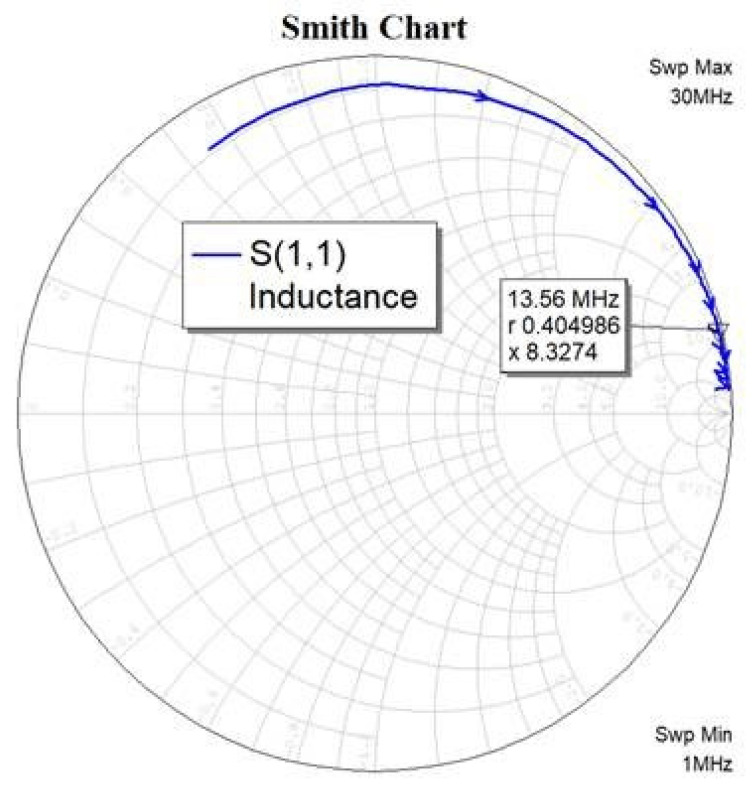
Smith chart and measured impedance for the fabricated flexible PCB antenna at 13.56 MHz.

Once fabricated, the antenna inductance was measured using a VNA, and the result can be seen in Figure 4. Converting the impedance, we find that the inductance of the antenna is indeed 4.88 µH.

### 2.3. Enclosure Design

**Figure 5:**
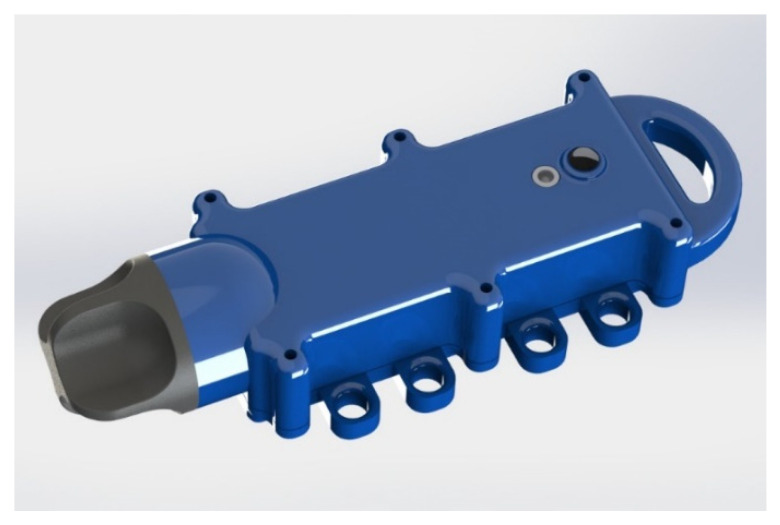
3D view of the enclosure designed for the device.

The enclosure was designed using the PCB 3D model and the battery size as base sizes. A screw-on cap is used to access the USB-C connector that is also waterproof. The back is designed with relief details to aid the attachment to the seaweed/kelp leaf using glue. The whole enclosure is waterproofed by using gaskets and screws, as well as using a polyurethane resin (PUR) inside the device to protect the electronics from water ingress. The holes on the sides provide a space to thread a line securing the device to the mooring lines in the farm. This is needed to make sure the device is not lost and becomes an environment pollutant unintentionally. The 3D design of the enclosure can be seen in Figure 5.

### 2.4. Firmware Design

The sensor device has three modes of operation: shutdown, sleep, and normal. During shutdown, all the components are turned off, except for the NFC chip, ON/OFF controller, and the RTC (real-time clock) on the microcontroller. The shutdown mode can be entered via sending a turn-off command to the device via either NFC or USB.

**Figure 6:**
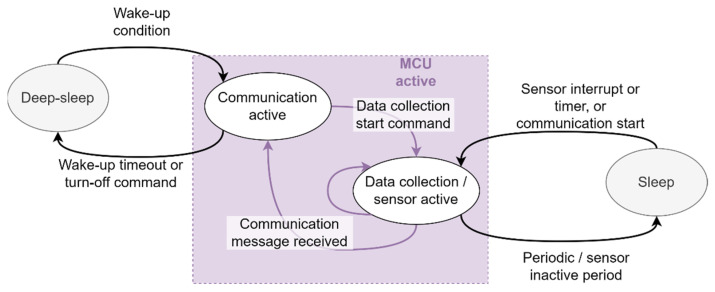
State diagram for seaweed sensor operations.

During normal operation, the device is either communicating with the USB host or the NFC reader, or the device is collecting sensor data. Otherwise, the device enters the sleep mode to save battery power while still waking up periodically to collect the sensor data. Figure 6 shows the modes of operation and the transition between these states.

**Figure 7:**
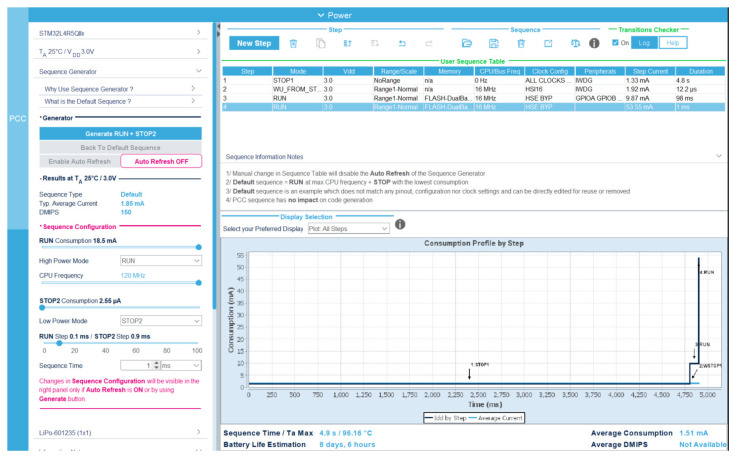
Power consumption profile calculated by STMCubeMX battery life estimator.

During data collection from the sensors, the read data are saved in a buffer inside the MCU memory. To reduce power consumption, the MCU only writes the data collected in the external flash memory when this buffer is full. In this way, the external memory is turned off for most of the time, and only turned on during transfers. If the device is configured to collect data, the sensor data collection subroutine should be running even while it is communicating with a host. To optimize the battery life, STM32CUBEMX battery life estimator has been used. To specify the timing requirements of the estimator, the clock counter of the data watchpoint and trace (DWT) unit of STM32 has been used. To minimize the battery power consumption, all non-essential peripherals were disabled, and the microcontroller was set to run at 16MHz to minimize the current consumption of the CPU. In Figure 7, the STM32CubeMX battery life estimator has been shown.

**Figure 8:**
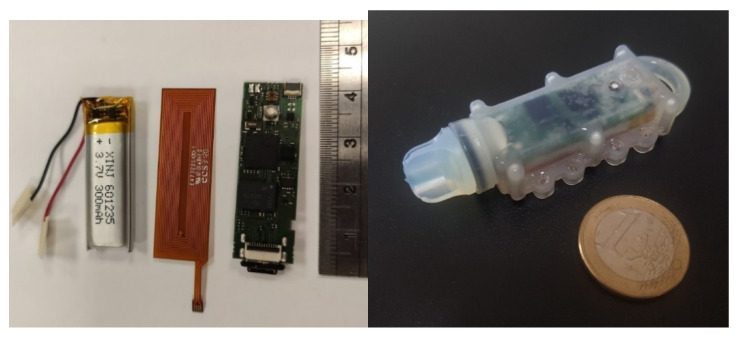
Final embedded system for seaweed monitoring before and after encapsulation for deployment.

The final embedded system developed is shown in Figure 8 with the individual component parts of the device: battery, motherboard, and NFC antenna, as well as the encapsulated system ready for deployment. From hereafter, this system will be called AquaBit.

### 2.5. Host Application

To communicate with the device once it is enclosed and to download data, two host applications were developed: (1) an Android application to be used in an NFC-enabled smartphone and (2) a Windows software run in a PC or laptop.

The Android app was developed using Android Studio with the help of the Software Development Kit (SDK) for the ST25DV [43] tag made available by STMicroelectronics. The application uses the SDK and the Android NFC libraries to communicate with the ST25DV tag using its custom commands according to its datasheet [40]. It can also communicate via the standard NFC Forum Type 5 standard commands as implemented by the SDK [43].

**Figure 9:**
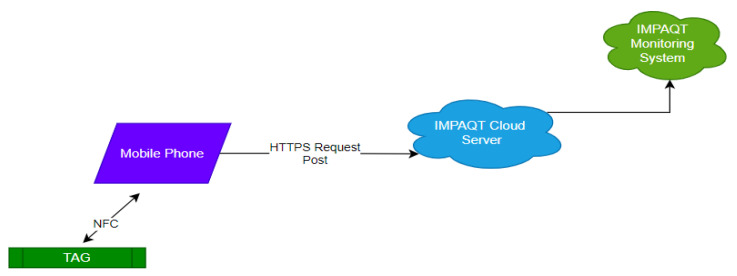
Data upload procedure to the IMS for visualisation.

The application functionality is as follows: the user places the smartphone close to the device with the application open. The application reads the device configuration and enables the user to change it by showing the options on the screen. The user has the option to download the logged data and upload them to the IMPAQT cloud severs, which can be used to visualize the data on the IMPAQT monitoring system (IMS). The uploading procedure is shown in Figure 9.

To communicate via USB, a Python 3.6 application was developed to work with the *pyusb* package [44] that provides a wrapper to USB functionality in Windows-32 environments. In the device side, the USB stack was set to function as a USB communications device class (CDC), using the library provided by STMicroelectronics for ST32L4 microcontrollers [45].

This Python application (hereafter called PyHost) is able to send commands to the device, update its internal real time clock, and download data from the device internal memory. It also has debug capabilities and can update the firmware of the device without the access to the programmer connector.

## 3. Results

The final embedded system has been fully characterised in Lab settings to ensure that accurate measurements and datasets will be obtained when deployed in the seaweed farm associated with the IMPAQT project [46].

A general functionality test was also performed to determine system usability. The achieved read range for the NFC communication was dependent on the reader: different smartphones used provided different read distances. The maximum achieved distance on the air was 5 cm.

### 3.1. Sensors Characterisation and Calibration

#### 3.1.1. Inertial Measurement Unit

To characterise and calibrate the Inertial Measurement Unit (IMU), we used a motion capture (MoCap) system and a pendulum device with a gimbal platform attached to it. The system was put in movement by oscillating the pendulum while the gimbal was set to a specific orientation, so all 3 axes of the IMU were at least once the focus of the oscillation.

**Figure 10:**
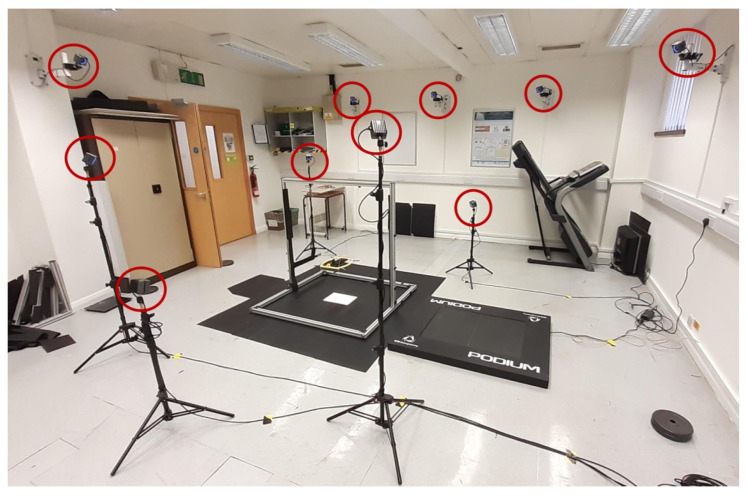
Positioning of the MoCap system cameras in relation to the device. Cameras are highlighted with red circles.

The motion capture (MoCap) system used consists of 10 infrared cameras by Optitrack (Prime^X^13, NaturalPoint, Inc. DBA Optitrack, Corvallis, OR, USA) [47] mounted in different positions, heights, and distance from the active area, pointed at the same area. The position of the cameras is shown in Figure 10. This system is designed to track the position of passive or active (IR-LEDs) markers placed in the object under observation and uses these markers to determine the position and orientation of the object in relation to global predetermined coordinates. Each camera has a 1.3 MP resolution, and its frame rate can be adjusted between 30 and 240 FPS. In this experiment, passive reflective markers of 12.5 mm diameter were used. The cameras are connected to a computer using the Motive 2.2 software [48] that combines the data from the cameras to recreate the movement of the object being tracked. The MoCap system was calibrated according to the instructions of the manufacturer [49]. The calibration file was saved, as it contains the calibration error necessary to assess the results.

**Figure 11:**
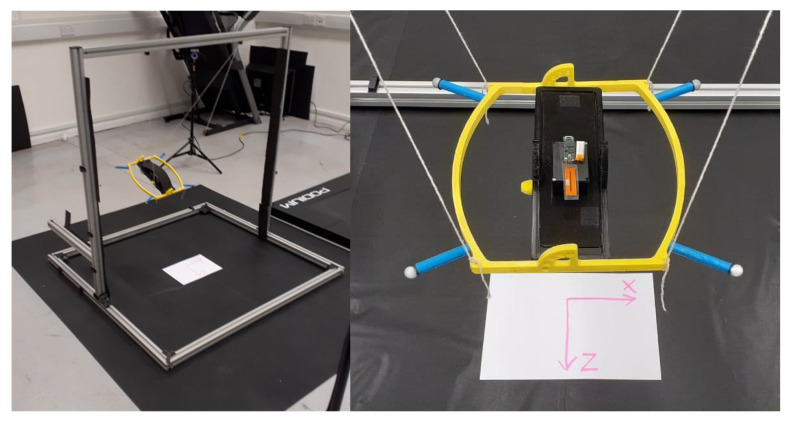
IMU test and calibration fixture developed for the characterisation.

The custom pendulum setup is composed of four strings attached to an aluminium rig structure via bolts and nuts and a gimbal platform with 2 DoF. Figure 11 shows the detailed photo of the gimbal platform where the device was placed with the passive reflective markers attached to the corners.

The procedure for the data capture was as follows:

1. The cameras’ FPS was set to be the same as the sampling frequency of the IMU (100 Hz).
2. The device was configured with the time and the IMU sampling frequency via the NFC Android application developed. Then, after placing the device in the specific orientation for the test, a start data recording command was sent to it.
3. In the Motive software, a recording was initiated, and the time was noted to be correlated with the data from the device.
4. The pendulum was pulled and then set free to oscillate until coming to a rest. This period was recorded in the Motive software and inside the device.
5. The data from the device were downloaded for comparison.

Data collected from the Motive system are the position of the object under observation in the Earth-fixed coordination frame, and data collected from the IMU sensor are accelerations in the X, Y, and Z directions in the sensor-fixed coordination frame. To compare these data, all of them must be mapped to a same coordination frame and they must represent a same physical quantity. Therefore, all collected data were mapped to a third coordination frame, body-fixed frame, and then, a second derivation of data collected from Motive system has been used as acceleration measured by Motive system.

**Figure 12:**
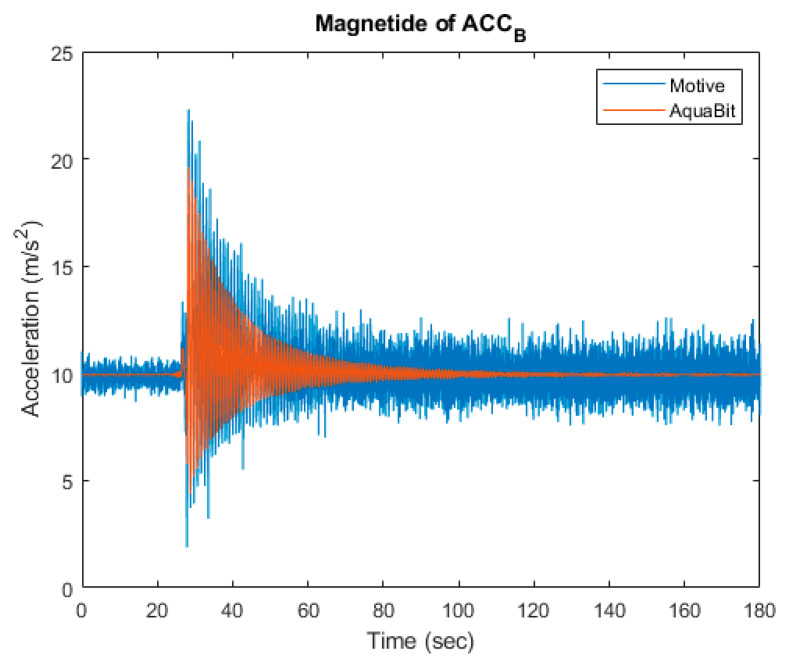
Time series of acceleration axis captured by the AquaBit device and the Motive MoCap system.

After the data collection, both the data exported from the Motive software and the data downloaded from the device were imported into MATLAB^®^ [50]. Both time series were synchronised and then compared against each other, as seen on Figure 12.

The accuracy of test results has been limited by the time synchronisation for AquaBit and the MoCap. The maximum error for the synchronisation is 1 s. Additionally, the resolution of time clock in the AquaBit used for the test was 4 ms. The sampling frequency used for both the AquaBit IMU and the MoCap system was 104 Hz. The accuracy achieved by calibrating the MoCap system for the experiments had a mean error of 0.6 mm.

The results logged from this test can then be used to calibrate the IMU using the method explained in Kim and Golnaraghi [51].

#### 3.1.2. Pressure Sensor

For the pressure sensor characterisation, the device was placed in a pressure vessel and a digital pressure gage (MTI DPGA12, Dwyer Instruments, Inc., Michigan City, IN, USA) monitored the pressure inside the vessel. The air pressure inside the vessel was increased in intervals of 5 psi (approximately 34.5 kPa) each 3 min, up to 30 psi (20.7 kPa)—which is approximately equivalent to a depth of 20 m. The pressure was set manually, and the sensor system was set to log the pressure.

**Figure 13:**
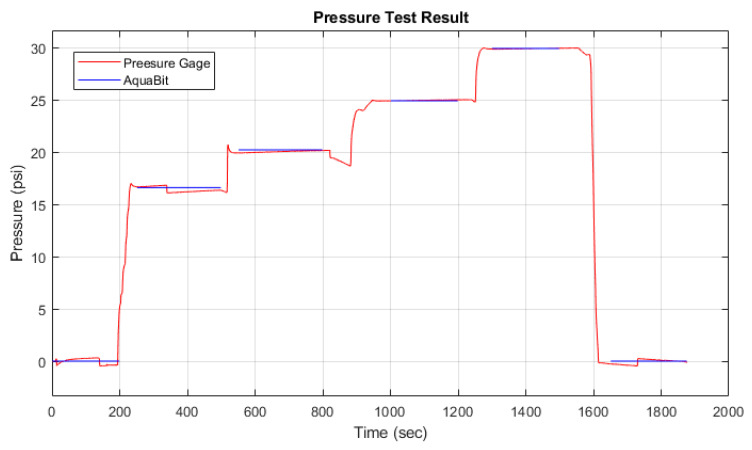
Pressure sensor test in air result.

In Figure 13, the blue line represents the pressure values measured by the gage, and the red line represents the measurements of AquaBit pressure sensor. As we can see, the difference between these two measurements is very low (max error is less than 6.9 Pa) in various pressures.

**Figure 14:**
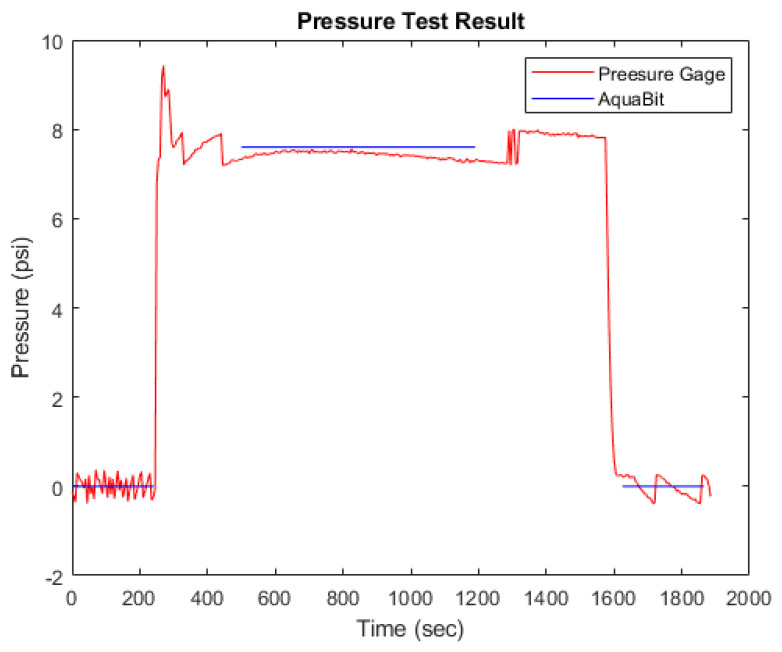
Pressure sensor test in water result.

**Figure 15:**
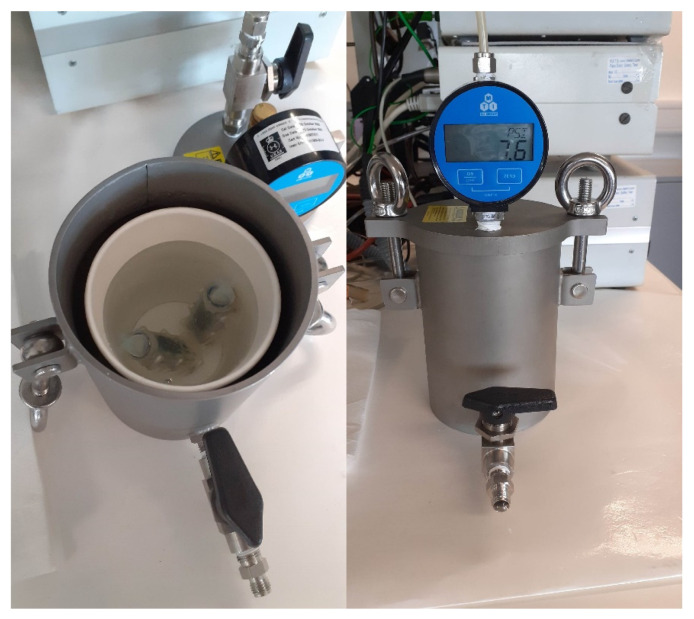
Test setup for the pressure sensor test in water.

In Figure 14, the result of the pressure test in water is presented. As shown, the difference between the blue lines, which represents the pressure measurements using a pressure gage, and the orange line, which represents the measurements using the pressure sensor on AquaBit, is very small. The max error is 0.5 psi (3.4 Pa), which approximately simulates 5 m depth in sea water. It is worth noting that measurements using the pressure gage have been done only when the pressure inside the pressure vessel was stable. The spike in the pressure measured by AquaBit between 200 s to 400 s is the normal overshoot that happened during adjusting the pressure. The setup used for this test is shown in Figure 15.

#### 3.1.3. Light Sensor

**Figure 16:**
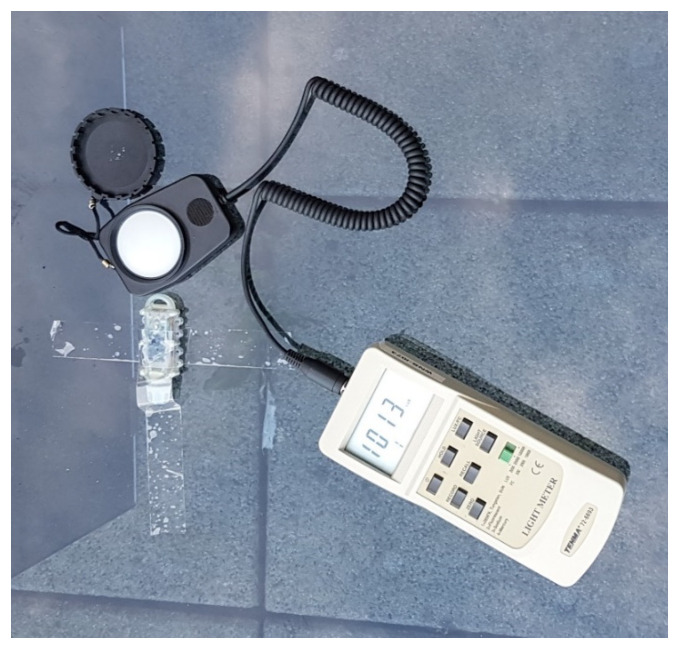
Light measurement calibration of AquaBit.

To characterise the light sensor, the device was placed in different natural light conditions with a lux meter. The value read by the lux meter was manually noted, and the device recorded the lux measurement read by the internal light sensor, as shown in Figure 16 below.

### 3.2. Power Consumption

**Figure 17:**
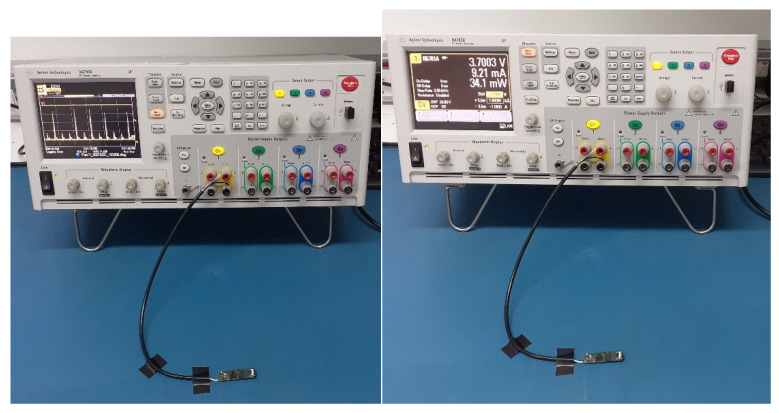
Power consumption measurement of battery lifetime using a DC power analyser.

Power consumption of the AquaBit has been measured using a power analyser (N6705B DC Power Analyser) to figure out how long the unit can do the sampling using its internal battery. As is shown in Figure 17, the power analyser was on the battery emulator mode and was connected to the battery port of the unit.

**Figure 18:**
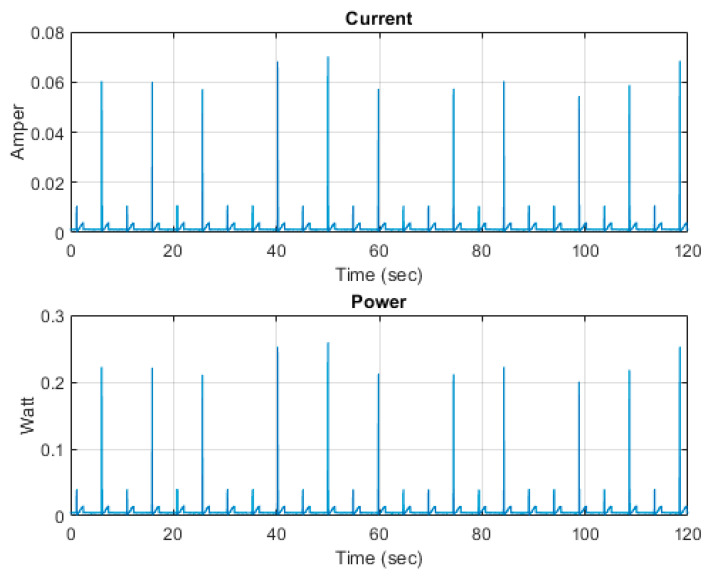
AquaBit current and power consumption during sampling.

The power consumption of AquaBit is very low (~1 mA) for most of the time, since the unit is in sleep mode and it goes to higher values during the sampling, as is shown in Figure 18. The average of current and power consumptions are 1.6 mA and 5.7 mW.

The battery lifetime for the AquaBit depends on the sampling frequency set for the sensors. In the case shown in Figure 18, the expected autonomy is of 1 week. However, for most deployment cases where temperature, depth, and light measurements are taken in bigger intervals (1 h or more) the expected lifetime can be expanded. This can be configured to allow for customization for each deployment and study type.

## 4. Discussion

Some of the most important parameters that influence seaweed growth are water quality, temperature, light radiation, water pH, nutrient availability, and wave incidence. For an IMTA site, it is necessary to monitor these parameters with as best resolution as possible to maximise production and minimise environmental impact. Current commercial and research seaweed monitoring technology can cover large areas, but for small-scale, fine-resolution monitoring, no integrated solution is available to the best of our knowledge. This work presents a solution that integrates multiple sensor modalities into a miniaturised package that can be deployed in multiple points of a farm so that fine-scale data can be collected and analysed.

The AquaBit device developed embeds a pressure sensor that can measure depth and temperature, a light sensor that measures incident radiation, and an IMU to track water movement and wave exposure. The data measured by these sensors are then logged internally in its memory and can be transmitted wirelessly via its NFC communication interface or wired USB connection. The device is rechargeable, reusable, and can be customised with different sampling frequencies to adapt to different needs.

All the sensors were characterised and calibrated in lab experiments. The results show that the sensors chosen were capable of measuring data according to the requirements established. Therefore, the device can be used to monitor environmental factors in a specific place in an aquaculture site, and the user can be sure that the data for each parameter are co-located and correlated with each other.

Since the device has an internal time keeping feature, the data collected are also dated to the second, which enables the user to plot the data in a time series and analyse it for trends and diel patters.

The device was waterproofed with the enclosure and the PUR rated for marine use. This means that the device can be deployed in either seawater or freshwater aquaculture farms.

The mechanical enclosure was designed to be flexible in the type of deployment, so the user can choose how to best deploy the device for their needs. Small eye holes on the sides allow for threading of lines to secure the device to mooring lines or to the seaweed itself. The backside has a pattern to provide a better surface area in case the user wants to glue the device to the seaweed/kelp blades. This type of deployment would enable the collection of data specifically about the wave and water motion effects on the seaweed. Since the device is very small, it does not add to the drag or additional stress to the seaweed.

As for communication, the USB interface works as expected. It can be used to communicate with the device, download data, and reprogram the device.

For the NFC communication, an Android app was developed to communicate with the device. It can send commands (such as wake-up, shutdown, start data collection, etc.), change the sensors configuration and sampling frequency, and download data. The range achieved for this wireless communication is not as big as expected. This could be because of the very small size of the antenna (limited by the device size), the presence of metal (circuit and battery) next to the antenna, and the unoptimized NFC reader antenna (smartphone). However, the system read range was sufficient to enable a data download using a standard smartphone with the required capability. A dedicated NFC reader with higher transmission power and bigger antenna could increase the read range.

An important feature of the device that we made sure was present is the customisability of the sensors sampling frequency. Especially in the case of the IMU, the sampling frequency greatly affects the type of data that can be extracted using data analysis methods. A high sampling frequency allows the user to detect very fast movements that would otherwise be missed. However, this needs to be balanced against power consumption, as the higher the sampling frequency, the higher the power consumption is and the less time the device can be deployed due to the battery lifetime.

The device was optimised for low-power consumption, from the hardware design to the firmware development, making sure multiple power options are available. To preserve battery while not deployed, the device has a hardware on/off controller that can put the system in shutdown mode or wake it up via the USB or the NFC interfaces.

As the power test shows, the device’s battery can last for a week if the sampling frequency is set to 1 Hz. A bigger period between samples would increase the time the device can be deployed, and this can be customised by the user.

### Future Work

The next step for this study is the deployment of the AquaBit device in a seaweed farm. This is currently in progress at the Marine Institute research facility [52].

Further improvements for the device include the development of embedded data analysis algorithms that can process data internally in the device and then transmit only the analysed results, thus minimising the size of transmitted data. The functionality for this is already present in the current version, as the microcontroller chosen has enough processing power to do so.

Another planed improvement is the addition of wireless charging that would greatly simplify the use and deployment of the device. Since the achieved transmission range of the NFC/RFID communication system designed for the device is small, changing the communication to Bluetooth may not reduce the achieved transmission range so much, but it would greatly increase the data rate and usability of the device. However, this option would greatly increase the power consumption of the device.

## Data Availability

No data.

## References

[1] Chopin T., Buschmann A.H., Halling C., Troell M., Kautsky N., Neori A., Kraemer G.P., Zertuche-González J.A., Yarish C., Neefus C. (2001). Integrating Seaweeds into Marine Aquaculture Systems: A Key Toward Sustainability. J. Phycol..

[2] Ridler N., Wowchuk M., Robinson B., Barrington K., Chopin T., Robinson S., Page F., Reid G., Szemerda M., Sewuster J. (2007). Integrated Multi—Trophic Aquaculture (Imta): A Potential Strategic Choice for Farmers. Aquac. Econ. Manag..

[3] Schroeder S.B., Dupont C., Boyer L., Juanes F., Costa M. (2019). Passive Remote Sensing Technology for Mapping Bull Kelp (Nereocystis Luetkeana): A Review of Techniques and Regional Case Study. Glob. Ecol. Conserv..

[4] Bennion M., Fisher J., Yesson C., Brodie J. (2019). Remote Sensing of Kelp (Laminariales, Ochrophyta): Monitoring Tools and Implications for Wild Harvesting. Rev. Fish. Sci. Aquac..

[5] Ottinger M., Clauss K., Kuenzer C. (2016). Aquaculture: Relevance, Distribution, Impacts and Spatial Assessments—A Review. Ocean Coast. Manag..

[6] Meneghesso C., Seabra R., Broitman B.R., Wethey D.S., Burrows M.T., Chan B.K.K., Guy-Haim T., Ribeiro P.A., Rilov G., Santos A.M. (2020). Remotely-Sensed L4 SST Underestimates the Thermal Fingerprint of Coastal Upwelling. Remote Sens. Environ..

[7] Woo H.-J., Park K.-A. (2020). Inter-Comparisons of Daily Sea Surface Temperatures and In-Situ Temperatures in the Coastal Regions. Remote Sens..

[8] Brewin R.J.W., Smale D.A., Moore P.J., Dall’Olmo G., Miller P.I., Taylor B.H., Smyth T.J., Fishwick J.R., Yang M. (2018). Evaluating Operational AVHRR Sea Surface Temperature Data at the Coastline Using Benthic Temperature Loggers. Remote Sens..

[9] Hurd C.L. (2000). Water Motion, Marine Macroalgal Physiology, and Production. J. Phycol..

[10] Visch W., Nylund G.M., Pavia H. (2020). Growth and Biofouling in Kelp Aquaculture (Saccharina Latissima): The Effect of Location and Wave Exposure. J. Appl. Phycol..

[11] Kregting L., Blight A.J., Elsäßer B., Savidge G. (2016). The Influence of Water Motion on the Growth Rate of the Kelp Laminaria Digitata. J. Exp. Mar. Biol. Ecol..

[12] Bekkby T., Rinde E., Gundersen H., Norderhaug K.M., Gitmark J.K., Christie H. (2014). Length, Strength and Water Flow: Relative Importance of Wave and Current Exposure on Morphology in Kelp Laminaria Hyperborea. Mar. Ecol. Prog. Ser..

[13] Burrows M.T., Harvey R., Robb L. (2008). Wave Exposure Indices from Digital Coastlines and the Prediction of Rocky Shore Community Structure. Mar. Ecol. Prog. Ser..

[14] Focht R.C., Shima J.S. (2020). Acceleration Loggers Reveal Fine-Scale Heterogeneity in Wave Exposure along an Open Coast. Estuar. Coast. Shelf Sci..

[15] Fan L.I.N., Meirong D.U., Hui L.I.U., Jianguang F.A.N.G., Lars A., Zengjie J.I.A.N.G. (2020). A Physical-Biological Coupled Ecosystem Model for Integrated Aquaculture of Bivalve and Seaweed in Sanggou Bay. Ecol. Model..

[16] Kerrison P.D., Stanley M.S., Edwards M.D., Black K.D., Hughes A.D. (2015). The Cultivation of European Kelp for Bioenergy: Site and Species Selection. Biomass Bioenergy.

[17] El Mahrad B., Newton A., Icely J.D., Kacimi I., Abalansa S., Snoussi M. (2020). Contribution of Remote Sensing Technologies to a Holistic Coastal and Marine Environmental Management Framework: A Review. Remote Sens..

[18] García-Poza S., Leandro A., Cotas C., Cotas J., Marques J.C., Pereira L., Gonçalves A.M.M. (2020). The Evolution Road of Seaweed Aquaculture: Cultivation Technologies and the Industry 4.0. Int. J. Environ. Res. Public Health.

[19] Evans S.N., Abdo D.A. (2010). A Cost-Effective Technique for Measuring Relative Water Movement for Studies of Benthic Organisms. Mar. Freshw. Res..

[20] HOBO Pendant Temperature/Light Data Logger 64K. https://www.onsetcomp.com/products/data-loggers/ua-002-64.

[21] Lyman T.P., Elsmore K., Gaylord B., Byrnes J.E.K., Miller L.P. (2020). Open Wave Height Logger: An Open Source Pressure Sensor Data Logger for Wave Measurement. Limnol. Oceanogr. Methods.

[22] Kennedy D., Cullen E., McNulty R., Gaffney M., Walsh M., O’Flynn B. (2015). Marine Inertial Measurement Units: Communication, Capabilities, and Challenges. Mar. Technol. Soc. J..

[23] Judge R., Choi F., Helmuth B. (2018). Recent Advances in Data Logging for Intertidal Ecology. Front. Ecol. Evol..

[24] IButton—IButton Devices—One Wire|Maxim Integrated. https://www.maximintegrated.com/en/products/ibutton-one-wire/ibutton.html.

[25] TidbiT v2 Temperature Data Logger—UTBI-001. https://www.onsetcomp.com/products/data-loggers/utbi-001.

[26] Knight P., Bird C., Sinclair A., Higham J., Plater A. (2021). Testing an “IoT” Tide Gauge Network for Coastal Monitoring. IoT.

[27] Beddows P.A., Mallon E.K. (2018). Cave Pearl Data Logger: A Flexible Arduino-Based Logging Platform for Long-Term Monitoring in Harsh Environments. Sensors.

[28] LTC2955 Datasheet and Product Info|Analog Devices. https://www.analog.com/en/products/ltc2955.html#.

[29] STM32L4R5/S5—STMicroelectronics. https://www.st.com/en/microcontrollers-microprocessors/stm32l4r5-s5.html.

[30] EEMBC—CPU/MCU Performance Benchmark—CoreMark. https://www.eembc.org/coremark/scores.php.

[31] EEMBC—CPU/MCU Energy Benchmark—ULPMark. https://www.eembc.org/ulpmark/ulp-cp/scores.php.

[32] Lambo L., Shengli Z., Jun-Hong C. (2008). Prospects and Problems of Wireless Communication for Underwater Sensor Networks. Wirel. Commun. Mob. Comput..

[33] Gussen C.M.G., Diniz P.S.R., Campos M.L.R., Martins W.A., Costa F.M., Gois J.N. (2016). A Survey of Underwater Wireless Communication Technologies. JCIS.

[34] Che X., Wells I., Dickers G., Kear P., Gong X. (2010). Re-Evaluation of RF Electromagnetic Communication in Underwater Sensor Networks. IEEE Commun. Mag..

[35] Domingo M.C. (2012). Magnetic Induction for Underwater Wireless Communication Networks. IEEE Trans. Antennas Propag..

[36] Peres C., Pigeon M., Rather N., Gawade D., Buckley J., Jafarzadeh H., O’Flynn B. (2020). Theoretical Models for Underwater RFID and the Impact of Water Salinity on the Design of Wireless Systems. Int. J. Adv. Netw. Serv..

[37] Finkenzeller K. (2014). RFID Handbook: Fundamentals and Applications in Contactless Smart Cards, Radio Frequency Identification and Near-Field Communication.

[38] Coskun V., Ozdenizci B., Ok K. (2013). A Survey on Near Field Communication (NFC) Technology. Wirel. Pers. Commun..

[39] European Computer Manufacturers Association (2004). ECMA340—Near Field Communication Interface and Protocol (NFCIP-1).

[40] STMicroelectronics ST25DV04K—Datasheet 2018. https://www.st.com/resource/en/datasheet/st25dv04k.pdf.

[41] NFC Antenna EDesignSuite—STMicroelectronics. https://eds.st.com/antenna/.

[42] STMicroelectronics AN2866—How to Design a 13.56 MHz Customized Antenna for ST25 NFC/RFID Tags 2020. https://www.st.com/content/ccc/resource/technical/document/application_note/d9/29/ad/cc/04/7c/4c/1e/CD00221490.pdf/files/CD00221490.pdf/jcr:content/translations/en.CD00221490.pdf.

[43] STSW-ST25SDK001—Software Development Kit for ST25 Tags and Dynamic Tags—STMicroelectronics. https://www.st.com/en/embedded-software/stsw-st25sdk001.html.

[44] PyUSB. https://pyusb.github.io/pyusb/.

[45] UM1734—STM32CubeTM USB Device Library User Manual 2019. https://www.st.com/resource/en/user_manual/dm00108129-stm32cube-usb-device-library-stmicroelectronics.pdf.

[46] IMPAQT Project. https://impaqtproject.eu/about-impaqt/.

[47] Primex 13—Specs. http://optitrack.com/cameras/primex-13/specs.html.

[48] Motive—Optical Motion Capture Software. http://www.optitrack.com/software/motive/index.html.

[49] Calibration—NaturalPoint Product Documentation Ver 2.2. https://v22.wiki.optitrack.com/index.php?title=Calibration.

[50] MATLAB—MathWorks. https://www.mathworks.com/products/matlab.html.

[51] Kim A., Golnaraghi M.F. Initial Calibration of an Inertial Measurement Unit Using an Optical Position Tracking System. Proceedings of the PLANS 2004. Position Location and Navigation Symposium (IEEE Cat. No.04CH37556).

[52] Our Ocean: Our Livelihoods|Marine Institute. https://www.marine.ie/Home/site-area/areas-activity/education-outreach/our-ocean-our-livelihoods.

